# Altered Brain Functional Network in Subtypes of Parkinson's Disease: A Dynamic Perspective

**DOI:** 10.3389/fnagi.2021.710735

**Published:** 2021-09-07

**Authors:** Junlan Zhu, Qiaoling Zeng, Qiao Shi, Jiao Li, Shuwen Dong, Chao Lai, Guanxun Cheng

**Affiliations:** ^1^Department of Radiology, Peking University Shenzhen Hospital, Shenzhen, China; ^2^Department of Radiology, National Cancer Center/National Clinical Research Center for Cancer/Cancer Hospital and Shenzhen Hospital, Chinese Academy of Medical Sciences and Peking Union Medical College, Shenzhen, China

**Keywords:** Parkinson's disease, functional magnet resonance imaging, functional connectivity, dynamic, graph theory

## Abstract

**Background:** Parkinson's disease (PD) is a highly heterogeneous disease, especially in the clinical characteristics and prognosis. The PD is divided into two subgroups: tremor-dominant phenotype and non-tremor-dominant phenotype. Previous studies reported abnormal changes between the two PD phenotypes by using the static functional connectivity analysis. However, the dynamic properties of brain networks between the two PD phenotypes are not yet clear. Therefore, we aimed to uncover the dynamic functional network connectivity (dFNC) between the two PD phenotypes at the subnetwork level, focusing on the temporal properties of dFNC and the variability of network efficiency.

**Methods:** We investigated the resting-state functional MRI (fMRI) data from 29 tremor-dominant PD patients (PDTD), 25 non-tremor-dominant PD patients (PDNTD), and 20 healthy controls (HCs). Sliding window approach, *k*-means clustering, independent component analysis (ICA), and graph theory analysis were applied to analyze the dFNC. Furthermore, the relationship between alterations in the dynamic properties and clinical features was assessed.

**Results:** The dFNC analyses identified four reoccurring states, one of them showing sparse connections (state I). PDTD patients stayed longer time in state I and showed increased FNC between BG and vSMN in state IV. Both PD phenotypes exhibited higher FNC between dSMN and FPN in state II and state III compared with the controls. PDNTD patients showed decreased FNC between BG and FPN but increased FNC in the bilateral FPN compared with both PDTD patients and controls. In addition, PDNTD patients exhibited greater variability in global network efficiency. Tremor scores were positively correlated with dwell time in state I along with increased FNC between BG and vSMN in state IV.

**Conclusions:** This study explores the dFNC between the PDTD and PDNTD patients, which offers new evidence on the abnormal time-varying brain functional connectivity and their network destruction of the two PD phenotypes, and may help better understand the neural substrates underlying different types of PD.

## Introduction

Parkinson's disease (PD) is a common progressive neurodegenerative disorder, characterized by tremor, rigidity, bradykinesia, and postural instability/gait disorders (Lees et al., 2009). Resting tremor is a core diagnostic feature affecting up to 70% of PD patients and a symptom relatively distinct from other motor signs of PD (Zetusky et al., 1985). Patients with PD can be grouped into tremor-dominant (TD) and non-tremor-dominant (NTD) phenotypes according to the presence of resting tremor or not (Helmich et al., 2012; Marras and Lang, 2013). Relative to the TD phenotypes, the NTD phenotypes suffer more from postural and gait problems and have greater risk of dementia, worse level of cognitive decline (Williams-Gray et al., 2007, 2009), higher sensitivity for depression (Dissanayaka et al., 2010), and increased executive control deficits (Wylie et al., 2012). Revealing the neural mechanisms underlying this heterogeneity is crucial to improve our knowledge of PD subtypes and to identify effective treatments. However, the neural basis for these disparate manifestations is not well-understood.

Previous functional neuroimaging research studies have demonstrated that these clinical differences of the two subtypes are reflected by a variety of cerebral functional alterations. Nuclear imaging studies reported lower striatal dopaminergic and glucose metabolism in NTD patients (Spiegel et al., 2007; Helmich et al., 2011). Impairment differences in the corticostriatal pathways and relevant neural network circuits have been reported between the two PD subtypes. Some task-related functional MRI (fMRI) studies revealed distinct activation paradigm in the striato-(cerebello-)thalamo-cortical circuits between the two subtypes along with reduced activity in the globus pallidus and prefrontal cortex in NTD patients (Lewis et al., 2011; Prodoehl et al., 2013; Zhang J. et al., 2015). Moreover, previous studies using independent component analysis (ICA), which identified brain regions that fluctuate in synchrony and constitute reproducible and reliable brain functional networks, found altered functional coupling between brain networks, including corticostriatal network (Karunanayaka et al., 2016), and within network between the two subtypes (Guan et al., 2017). However, the majority of these earlier studies were on the basis of static functional connectivity, supposing that fluctuation of brain signals throughout the entire scan is constant, and the dynamic brain network changes and the mechanism of the two PD phenotypes are not yet clear.

The dynamic functional connectivity (dFC) investigates the time-varying brain communication by measuring changes in the strength or spatiotemporal distribution of functional connectivity over time among brain regions (Hutchison et al., 2013). It provides a new viewpoint of abnormal dynamic brain communication and could capture FC changes motivated by disease pathophysiology (Preti et al., 2017; Khambhati et al., 2018). Clinical studies of multiple diseases including PD have suggested the potential biomarker utility and clinical relevance of dFC. For example, compared with controls, the proportion of segregated and integrated FNC states in PD patients appears obviously imbalanced, and the dynamic variables of FNC are closely related to the severity of clinical symptoms (Kim et al., 2017). Another work demonstrated that the intra-network variability of functional connectivity in salient, visual, and subcortical network was greater in PD patients, while that along with the inter-network variability presented a distributed variation (Zhu et al., 2019). Furthermore, the variability of brain network efficiency based on the graph theory analysis method combined with dFC may also provide important insight into the underlying neural substrate of PD. In addition, a recent study found that the variability of global efficiency over network in PD patients was higher, implying abnormal functional network integration of PD (Kim et al., 2017). However, alterations of the dynamic brain communication characteristics between the two PD phenotypes are yet poorly understood. From the dynamic perspective, we may help capture the abnormal functional alternation underlying two PD phenotypes that cannot be fully elucidated by static analysis methods.

Therefore, we intend to compare the properties of dFNC between the two PD phenotypes at the subnetwork level, using the FNC state analysis and graph theory analysis. This study aimed to (1) identify changes in dFC and in network topological characterization between TD and NTD phenotypes, (2) investigate whether the dFNC changes could characterize the potential discrepancy between the two PD phenotypes, and (3) explore whether these changes are associated with the clinical manifestation.

## Materials and Methods

### Subjects

In this study, we used data from the Parkinson's Progression Marker Initiative database (PPMI database, www.ppmi-info.org/data). PPMI is an ongoing multicenter cohort study with PD, and normal controls aimed at identifying biomarkers of PD progression; 54 patients with PD (29 tremor-dominant, PDTD and 25 non-tremor-dominant, PDNTD) and 20 healthy controls (HCs) were enrolled in our study between July 1, 2012, and November 29, 2016. Inclusion and exclusion criteria have been published in Parkinson Progression Marker Initiative (2011). For both PD patients and the controls, those without rs-fMRI images were excluded. PD patients were divided into two groups according to the Movement Disorder Society Unified Parkinson's Disease Rating Scale (MDS-UPDRS) part III of the resting tremor score (Prodoehl et al., 2013; van Nuland et al., 2020). A PDTD patient has an MDS-UPDRS-III resting tremor score of greater than two points for at least one limb, with a history of tremor. A PDNTD patient has an MDS-UPDRS-III resting tremor score of zero, without a history of tremor. The HC subjects were demographically comparable with the PD patients.

### Data Acquisition and Pre-Processing

All participants underwent resting-state MRI scans on 3.0T MRI scanners. A gradient-echo T2^*^-weighted echo-planar imaging sequence (GE-EPI) was applied to image brain functional activity with 210 time points during scanning. The fMRI acquisition parameters were as follows: time of repetition = 2,400 ms, echo time =25 ms, field of view = 224 mm, flip angle = 80°, voxel size = 3.3 mm^3^, and 40 axial slices.

Preprocessing of fMRI data was carried out using GRETNA software (http://www.nitrc.org/projects/gretna/). To achieve the equilibration of MRI signals and the adaptation of subjects to the scanning environment, the first 10 time points were discarded. The fMRI images of the remaining 200 time points were then slice-time corrected and realigned to the mean echo-planar image. Furthermore, all corrected fMRI images were spatially normalized into the Montreal Neurological Institute (MNI) standard space using EPI templates, resampled with a 3 mm isotropic voxel, and spatially smoothed with a 4 mm 3D-Gaussian filter. Three control patients with head motion exceeding 3 mm of translation motion or 3 degrees of rotation and 10 PD patients with poor-quality images were excluded from the analysis.

### Identification of Independent Components

The preprocessed fMRI data were broke down into independent components (ICs) *via* a group-level ICA carried out in the Group ICA of fMRI Toolbox (GIFTv3.0b, http://mialab.mrn.org/software/gift/). First, two data reduction steps were performed to decrease computational complexity. We cut down the subject-specific data with principal components analysis (PCA) followed by decomposition of the concatenated subject-reduced data in the group level including both the patient and control groups. Second, 39 ICs were automatically estimated through Group ICA utilizing the Infomax algorithm. Finally, the spatial functional networks for each subject and corresponding time courses were created using the ICA back reconstruction method.

**Figure 1:**
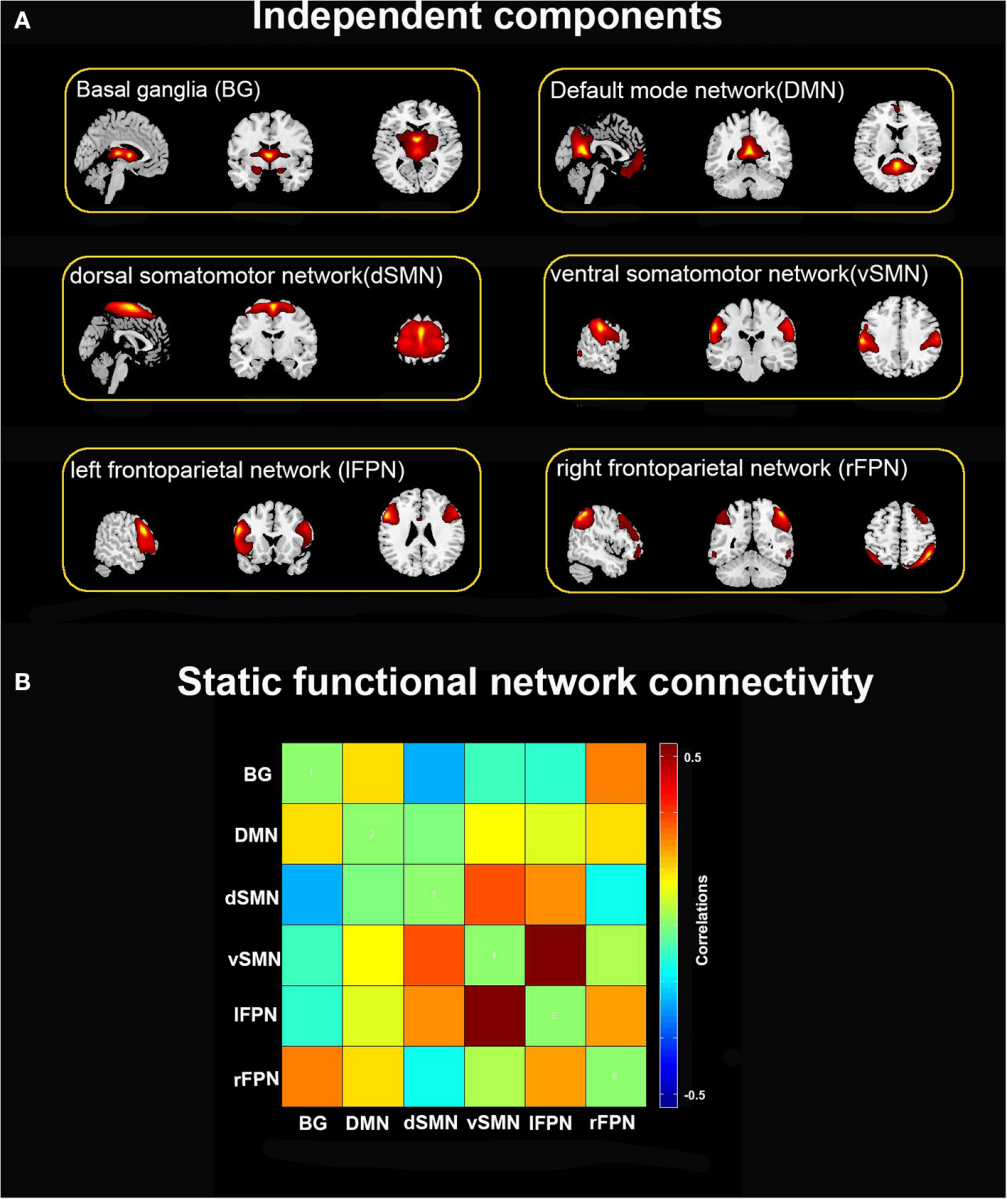
**(A)** Six independent functional components derived from the group ICA: basal ganglia (BG), default mode network (DMN), dorsal somatomotor network (dSMN), ventral somatomotor network (vSMN), left frontoparietal network (lFPN), and right frontoparietal network (rFPN). **(B)** Group averaged static functional connectivity matrix between six ICs.

Of the 39 ICs, 6 ICs of interest were identified that exhibited peak activations mostly in gray matter and higher low-frequency spectral power with low spatial overlap with known vascular, ventricles, cerebral white matter, and edge regions (Allen et al., 2014). Those ICs satisfying the expectations that ICs of interest should have the strongest anatomical correlation with the template were selected. Six ICs were characterized as subnetworks: basal ganglia (BG), default mode network (DMN), dorsal somatomotor network (dSMN), ventral somatomotor network (vSMN), left frontoparietal network (lFPN), and right frontoparietal network (rFPN), as shown in Figure 1.

Finally, we applied post-processing steps to eliminate noise, mainly including (a) detrending (linear, quadratic, and cubic), (b) analyzing the six realignment parameters by multiple regression, (c) despiking time courses, and (d) low-pass filtering (*f* < 0.15 Hz).

### Dynamic Functional Network Connectivity

We adopted the *k*-means clustering integrated with a sliding window approach in GIFT software to calculate the dFNC (Allen et al., 2014; Kim et al., 2017). To detect changes in FNC matrix across time, we created a sliding window of 48 s (20 TRs width) with a Gaussian value (σ = 3TRs) in steps of 1 TR, resulting in 180 sliding windows per subject. We chose the window length of 48 s because window size around 30–60 s could optimize the balance between the temporal resolution and the quality of the FNC estimate (Keilholz et al., 2013; Li et al., 2014). Each window of individual dFNC matrix was obtained using the values of pairwise Pearson's correlation between BOLD time courses of six ICs, resulting in time series of FNC matrixes (6×6) for subsequent analyses.

Next, all dFNC windows for each subject were classified by applying the *k*-means clustering based on the Euclidean distance, and the algorithm was repeated 100 times to reduce the bias of random initialization of centroid positions (Friedman et al., 2008). Four (*k* = 4) was determined as the optimal cluster using the elbow criterion. Consequently, three different variables, namely, mean dwell time, fractional windows, and number of transitions, were assessed to examine the temporal properties of dFNC states. Briefly, the mean dwell time was measured by averaging the quantity of consecutive windows in a specific state. The fractional windows referred to the percentage of time spent in one state. Accordingly, the number of transitions represented the number of times the conversion occurred from one state to another. Group comparisons were examined on functional connectivity strengths and temporal properties of FNC states using the two-sample *t*-test, followed by false discovery rate correction (FDR, *P* < 0.05).

### Graph Theory Analysis

We performed the graph theory analysis using the GRETNA software to analyze the variability of topological characterization of the FC network based on the ICs resulting from the above analysis. Herein, ICs corresponded to nodes, and connectivities linking nodes-pairs were defined as edges in the graphs (Rubinov and Sporns, 2010). We binarized all FNC matrixes and set a wide range of sparsity thresholds (0.1–0.5, with an interval of 0.05). As for the minimum sparsity selection, we applied a simple algorithm that was *S* = 2^*^log(*N*)/*N*−1, where *N* denoted the number of subjects. Global and nodal network indicators were calculated under the area of the curve (AUC) over the sparsity range, and AUC was widely applied in the graph theory analysis and proved to be sensitive to alterations in the topological organization of neurological diseases (He et al., 2009; Koshimori et al., 2016; Zhi et al., 2018). We studied the global efficiency and local efficiency as global and nodal network indicators, respectively. Global efficiency evaluated the capability of incorporating information of the whole network, while local efficiency characterized the fault tolerance of a network when a node was removed. Then, for every subject, the variance of the time-varying network efficiency was calculated. Graph-theoretical parameters analysis was assessed by one-way ANOVA and adjusted with FDR correction (*P* < 0.05) to detect the between-group differences.

### Statistical Comparisons and Correlations Analysis

Quantitative variables were analyzed *via* one-way ANOVA with pairwise *t*-test or Kruskal–Wallis one-way ANOVA with pairwise Mann–Whitney *U*-test according to its distribution. Qualitative variables were analyzed using chi-square test. The FDR correction for multiple comparisons was applied with *P* < 0.05. Additionally, Spearman's correlation analysis was performed to examine the relationship between altered dFNC metrics (functional connectivity strengths, temporal properties of FNC states, and network topological characterization) and clinical variables in PD patients. The clinical variables included resting tremor score, total tremor score, and MDS-UPDRS-III score. Since age, gender, and head motion could be modifiers of functional connectivity, nonetheless in our analysis, they were taken as covariates. Statistical analyses were performed utilizing SPSS version 26.0, and *P* < 0.05 was the threshold selected to be statistically significant.

To assess the diagnostic ability of dFNC in differentiating PDTD from PDNTD patients, univariate receiver operating characteristic (ROC) curves analysis was computed through SPSS software, calculating the area under the curve (AUC), sensitivity, and specificity. Here, we select the mean dwell time in state I as the most discriminating feature in ROC analysis.

## Results

### Demographic and Clinical Characteristics

Table 1 lists the detailed demographic and clinical characteristics of the PDNTD and PDTD patients, and controls. No significant differences were found in age, gender, or years of education between the three groups, except for MDS-UPDRS-III score and tremor score. The PDNTD group had significantly lower MDS-UPDRS-III score and tremor score than the PDTD group (*P* < 0.05).

**Table 1:** Characteristics of participants.

	**PDTD (*n* = 29)**	**PDNTD (*n* = 25)**	**Controls (*n* = 20)**	***p-*value**
Age (years)^a^	63.96 ± 7.73	59.36 ± 9.87	64.40 ± 8.07	00.08
Gender (female/male)^b^	13/16	8/17	04/16	00.20
Education (years)^c^	16 (15–18)	16 (14–18)	16.5 (16–18)	00.41
Duration of illness (years)^e^	3 (2–5)	3 (2–4)	–	00.37
Onset age (years)^d^	60.55 ± 8.04	56.44 ± 9.67	–	00.10
MDS-UPDRS III^d^	25.48 ± 8.77	18.84 ± 10.42	–	00.01^**^
Hoehn-Yahr stage^e^	2 (2–2)	2 (1–2)	–	00.12
Resting Tremor score^d^	2.86 ± 1.03	0.00 ± 0.00	–	<0.001^**^
Action/posture Tremor score^d^	1.86 ± 1.75	0.56 ± 1.00	–	0.001^**^

*Values are presented as mean ± SD or median (interquartile range 25–75%)*.

** *P < 0.05*.

MDS-UPDRS III, motor section of the Movement Disorder Society Unified Parkinson's Disease Rating Scale.

a *ANOVA*.

b *Pearson's χ^2^-test*.

c *Kruskal–Wallis test*.

d *Two-sample t-test*.

e *Mann–Whitney U-test*.

### Intrinsic Connectivity Networks

Figure 1A depicts the spatial map of the selected ICs using the Group ICA. According to their anatomical and presumed functional properties, the ICs were classified into the following networks: basal ganglia (BG), default mode network (DMN), dorsal somatomotor network (dSMN), ventral somatomotor network (vSMN), left frontoparietal network (lFPN), and right frontoparietal network (rFPN). Figure 1B shows the resting-state FNC matrix between ICs, which was averaged over all subjects.

### Dynamic Functional Connectivity States and Connectivity Strength

**Figure 2:**
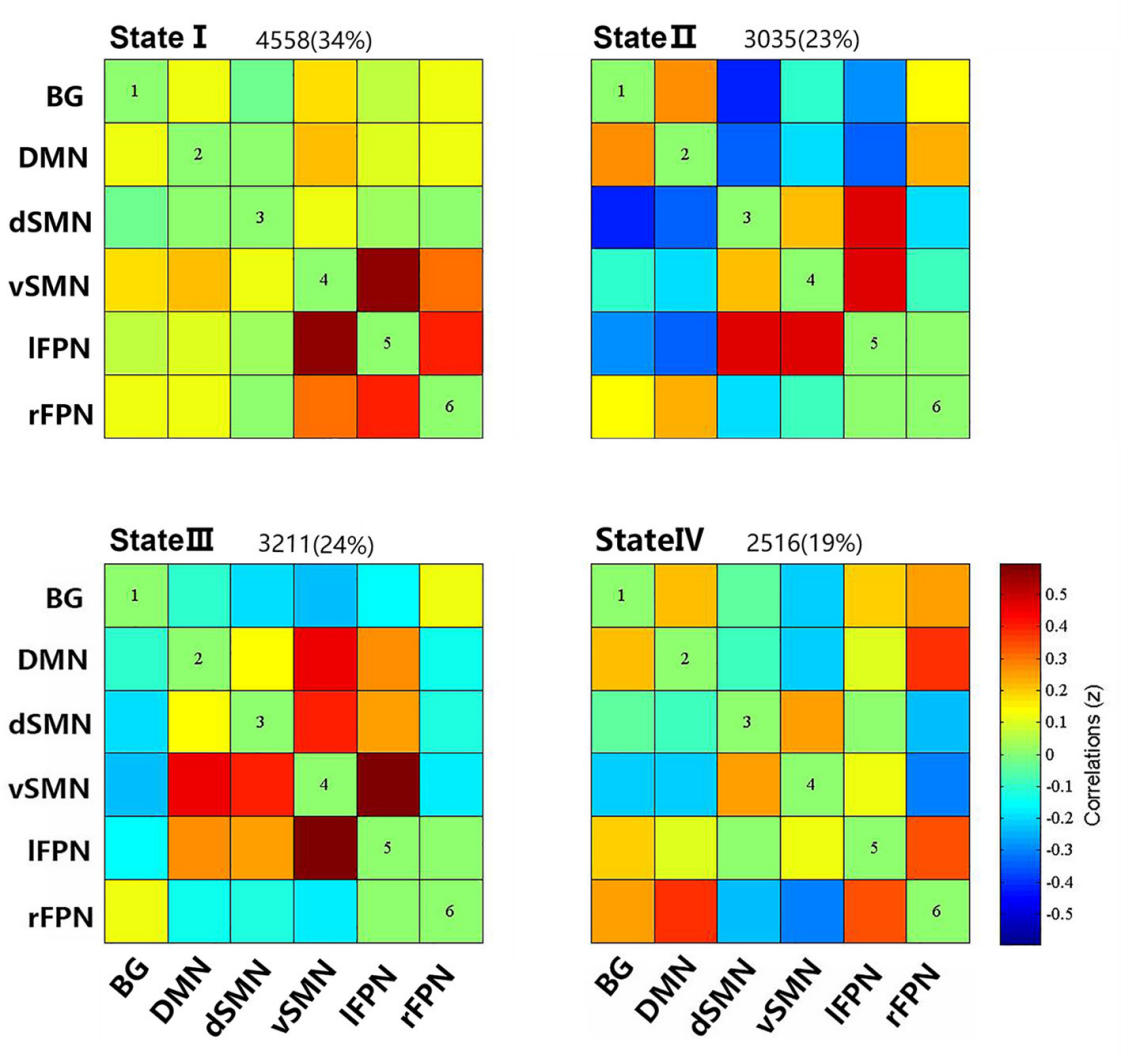
Cluster centroids of each states, and the total number and percentage of occurrence of the each brain connectivity state across the sliding windows of all subjects.

Four reoccurring states of dFNC matrixes were obtained throughout scans based on *k*-means clustering algorithm. The cluster centroid of the four states and their respective occurrence frequency and percentage are shown in Figure 2. With the above four cluster centroids, all dFNC windows of each subject were then divided into one of the four states based on their similarity to the cluster centroids; however, not all subjects have each of the four states. Each cluster centroid presents its corresponding connectivity patterns. State I occurred most frequently in four states (34%) and showed generally weak connectivity between ICs, except for strong and positive correlations between the vSMN and lFPN, and between the rFPN and lFPN. In contrast, the other three states demonstrated positive and negative couplings between ICs and were less frequent (state II 23%, state III 24%, and state IV 19%). State III distinguished itself from state II and state IV by the domination of a strong and positive connectivity of vSMN with DMN, dSMN, and lFPN. In state II, lFPN had a strong and positive connectivity with vSMN and dSMN but a negative connectivity with DMN, and dSMN had a negative connectivity with both BG and DMN. State IV was characterized by the presence of relatively strong connectivity related to rFPN, positive correlation with DMN and lFPN, and anti-correlations with vSMN.

**Figure 3:**
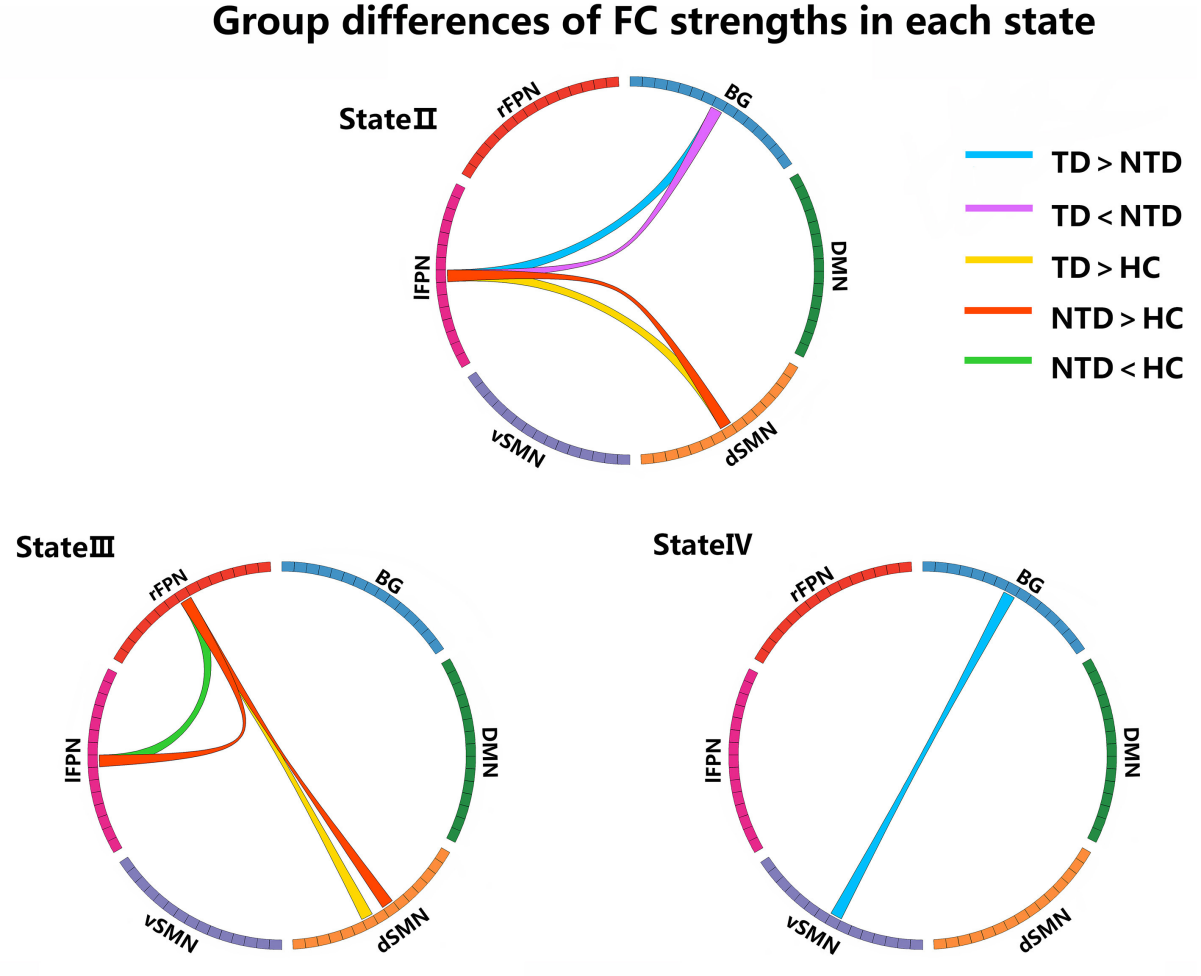
Group differences of FC strength in each state. Each square color represents one of the six networks. Blue lines represent increased connectivity, while purple lines represent decreased connectivity in PDTD patients compared with PDNTD patients. Yellow lines represent increased functional connectivity relative to controls. Red lines represent increased functional connectivity, while green lines represent decreased connectivity in PDNTD patients compared with controls. (*P* < 0.05, FDR corrected).

We compared the group differences of functional connectivity strengths among the triple groups in each state (*P* < 0.05, FDR corrected), as shown in Figure 3. Compared with HCs, both the PD groups showed higher FNC strengths between dSMN and lFPN in state II, and between dSMN and rFPN in state III. Two abnormal FNC strengths were observed in PDNTD patients relative to HCs, including reduced BG-lFPN junction in state II and stronger rFPN-lFPN junction in state III. Similarly, the two FNC differences were also found between PDTD and PDNTD patients. In particular, PDTD displayed higher strength in BG-lFPN junction than that in PDNTD, while lower strength in rFPN-lFPN junction. In addition, compared with PDNTD patients, we found greater FNC strength between BG and vSMN in state IV in PDTD patients. For state I, no significant differences were found between any other groups.

**Figure 4:**
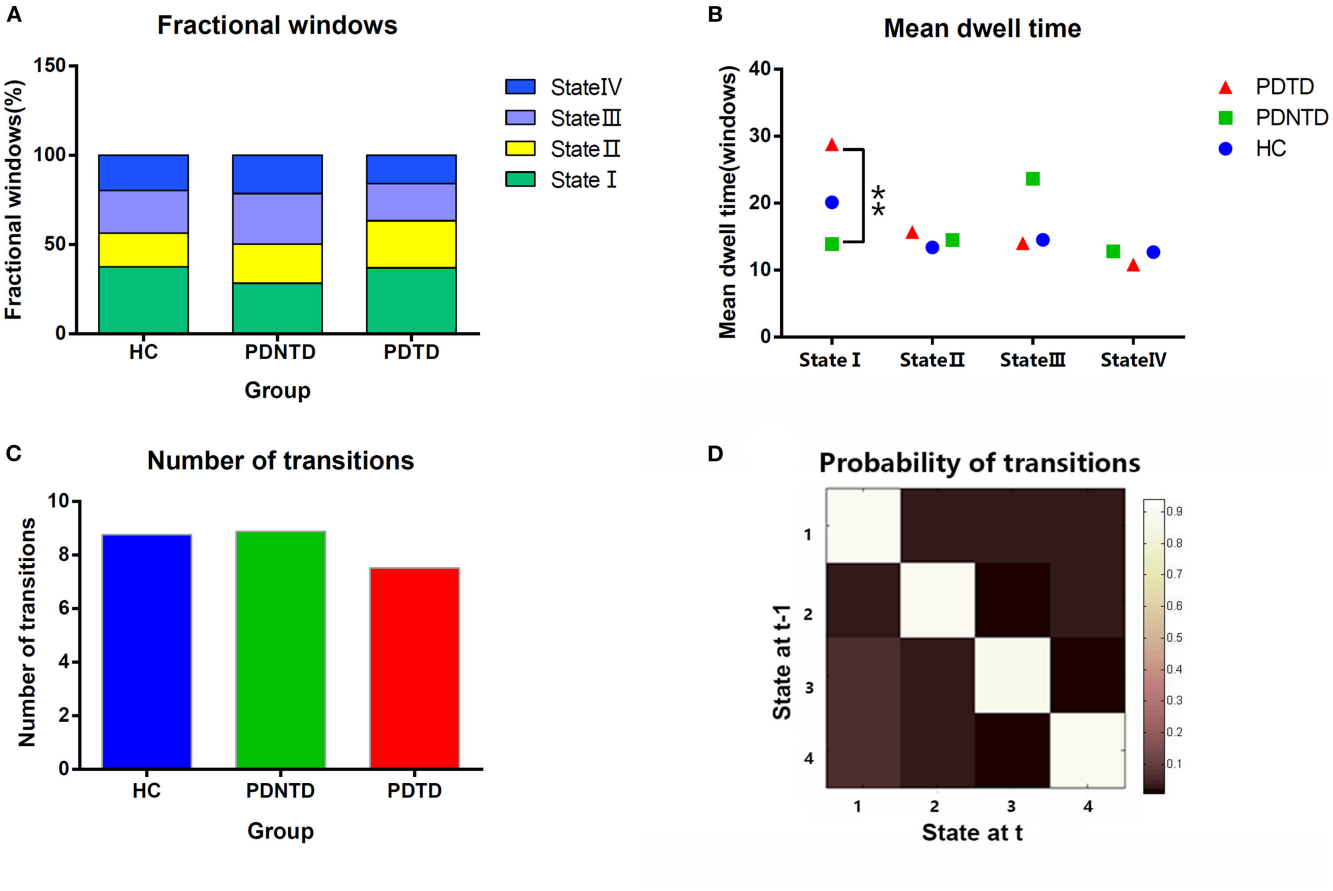
Comparison of group effect in the temporal properties of functional connectivity state among the PDTD, PDNTD, and control groups (*P* < 0.05, FDR corrected). **(A)** Fraction windows, **(B)** mean dwell time, and **(C)** number of transitions. **(D)** The state transition probability matrix, averaged over subjects. High values indicate a high probability of staying in a state. Asterisks represent significant differences at *P* < 0.05.

With regard to temporal properties, the mean dwell time in state I was significantly increased in PDTD patients compared with the PDNTD patients (*P* < 0.05, FDR corrected). However, there was no significant group effect either in fractional windows or in transition number, as shown in Figures 4A–C.

### Graph Topological Properties

**Figure 5:**
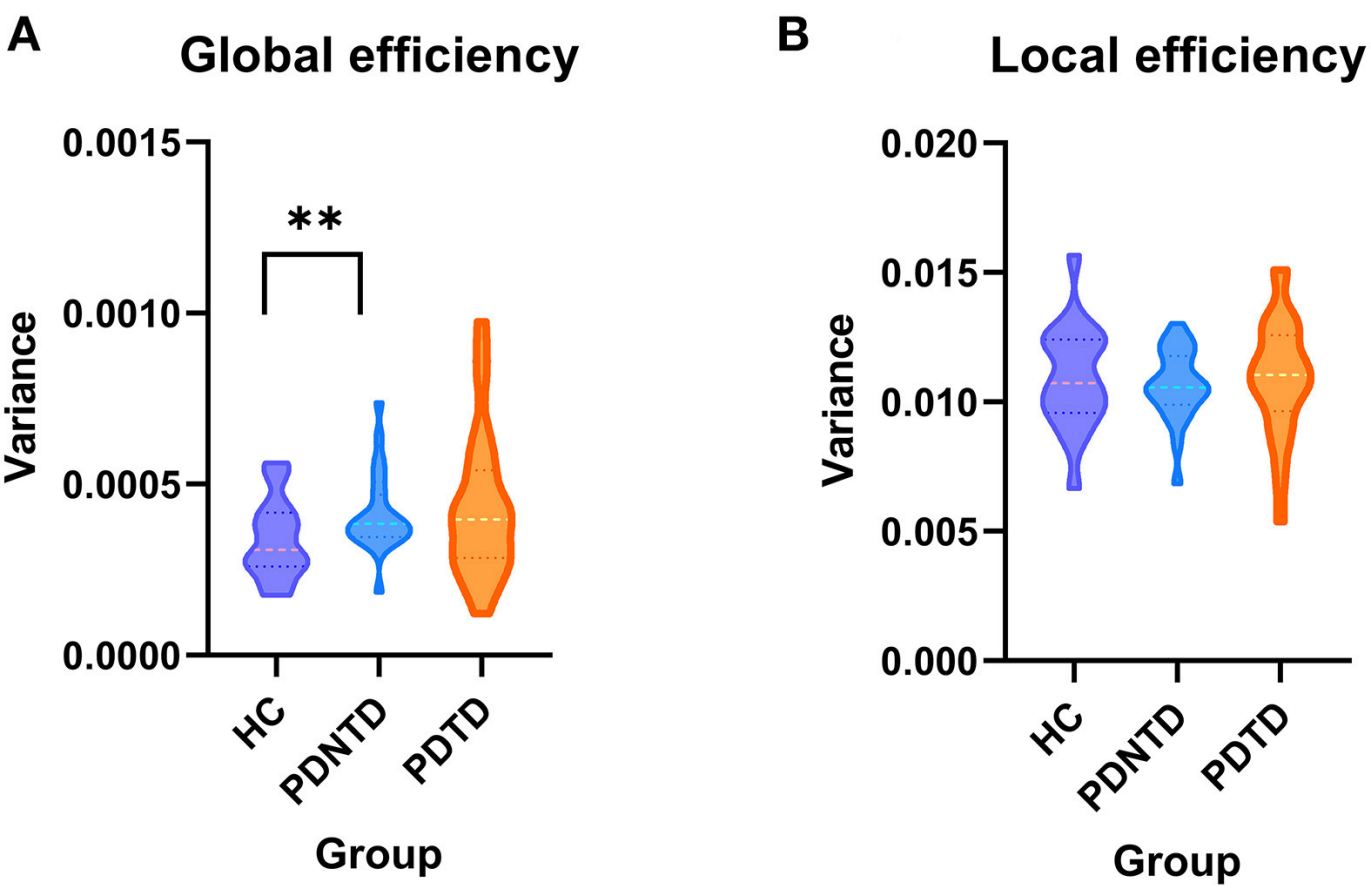
Between-group differences in the variances of network efficiency. The variances of **(A)** global efficiency and **(B)** local efficiency shown with violin plots. Asterisks represent significant differences at *P* < 0.05.

In terms of network topological metrics, we found increased variability of global efficiency in the PDNTD patients relative to the controls. In contrast, PD subtypes and HCs did not differ with regard to local efficiency. Figure 5 depicts the variability of global efficiency and local efficiency for each group.

### Relationship Between dFNC Properties and Clinical Characteristics

**Figure 6:**
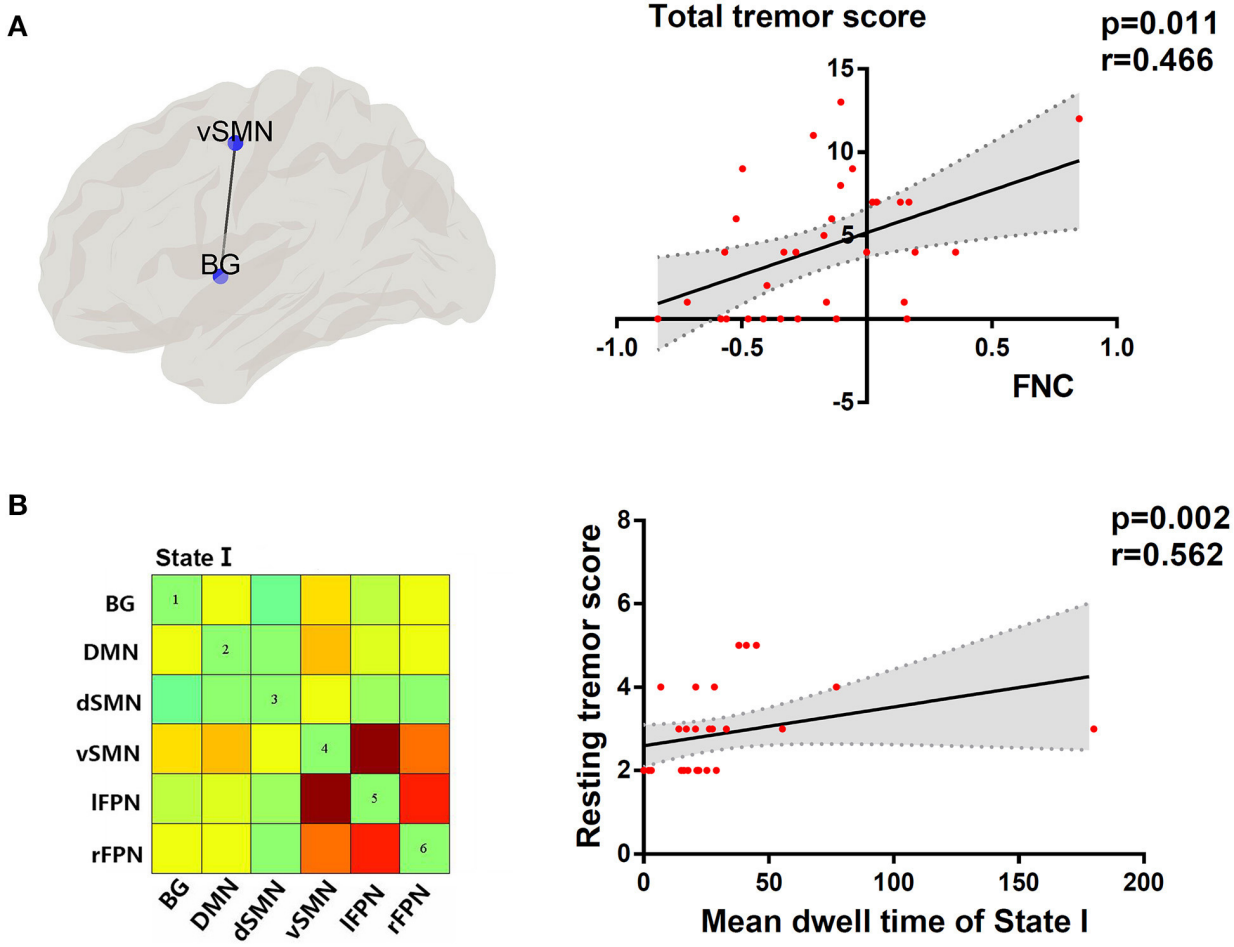
**(A)** Topographic representation of the connectivity between BG and vSMN. Correlation between total tremor score and FNC between BG and vSMN in state IV. **(B)** Cluster centroid of state I. Correlation between resting tremor score and mean dwell time in state I. Linear regression line with 95% CI for best-fit line (shading area), and *r* and *P*-values (Spearman's correlation coefficient) are provided.

As shown in Figure 6, the dFNC properties were significantly correlated with tremor symptom severity in the PD group with FDR-corrected for multiple comparisons. To be specific, the dwell time in state I was positively associated with resting tremor (*P* < 0.05, *r* = 0.562), suggesting that a higher percentage of time spent in the more sparsely inter-network connected was associated with poor tremor performance. In addition, the FNC strength between BG and vSMN in state IV had a positive correlation with the total tremor score, an item comprising resting tremor, postural tremor, and kinetic tremor (*P* < 0.05, *r* = 0.466). This indicated that greater the FNC strength of BG-vSMN coupling in state IV, the patients would have worse tremor performances.

**Figure 7:**
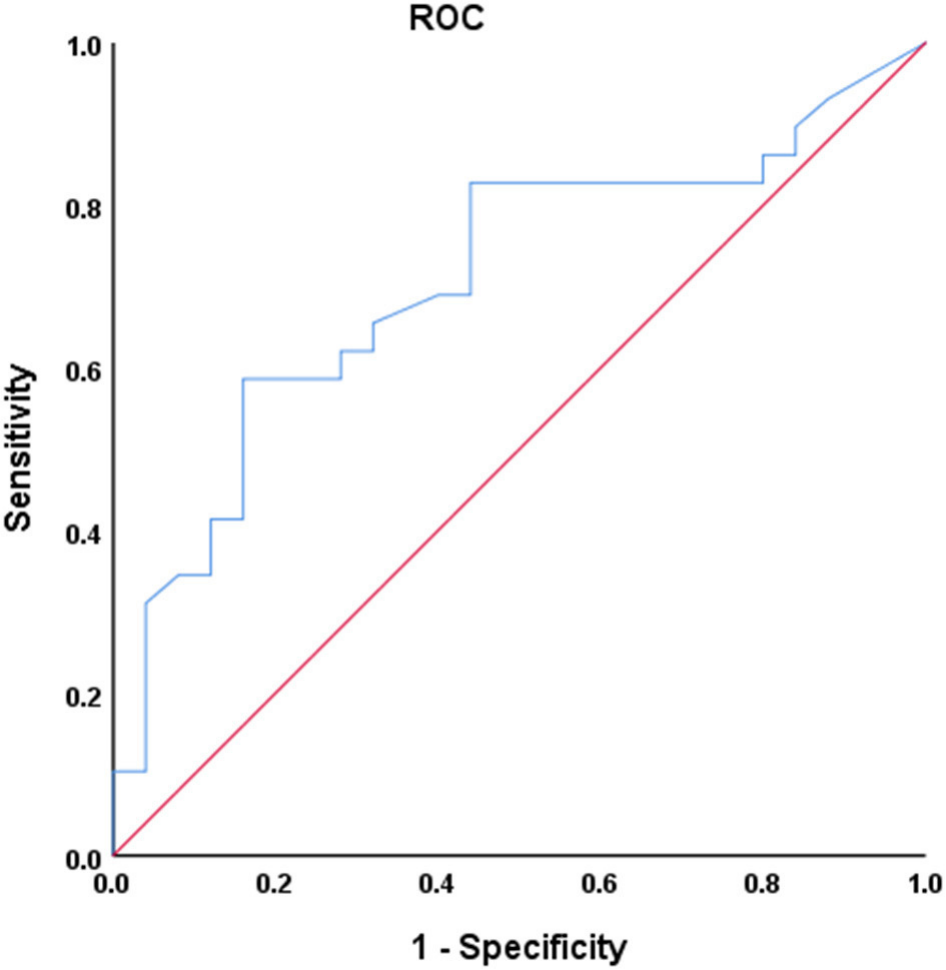
Receiver operating characteristic (ROC) curve of mean dwell time in state I for discriminator of PD phenotype. AUC = 0.709 [95% CI 0.569–0.849], *P* = 0.004, optimal cutoff value = 20.4, sensitivity = 0.586, specificity = 0.840.

Univariate ROC curves analysis in Figure 7 shows that the diagnostic value of mean dwell time in state I for PD phenotypes was relatively high: AUC = 0.709 [95% CI 0.569–0.849], optimal bound value = 0.426, sensitivity = 0.586, and specificity = 0.840.

## Discussion

In this study, we investigated the abnormalities of brain functional network in the two PD subtypes from a dynamical point of view, building on a combination of temporal properties of FNC and variability of network topological organization. The main findings were as follows: (1) Relative to PDNTD patients, PDTD patients spent more time in state I showing sparse connections, and the dwell time in state I was positively correlated with the resting tremor score; (2) PDTD patients and PDNTD patients exhibited altered dFNC strength between networks encompassing BG, vSMN, dSMN, l_FPN, and r_FPN. Meanwhile, the dFNC between BG and vSMN in state IV displayed a significant positive correlation with the total tremor score; and (3) PDNTD patients showed higher variability in global network efficiency compared with controls. Collectively, our findings provide new evidence that PDTD and PDNTD subgroups represent differential aberrant time-varying brain connectivity patterns.

### Increased Mean Dwell Time of the Sparsely Connected State in PDTD Patients

For what concerns the temporal properties, the dwell time in state I was discovered to provide good discrimination between the PDTD and PDNTD patients. PDTD patients stayed longer in the pattern of sparse connected state I relative to the PDNTD patients, mirrored by the increase in mean dwell time. Moreover, there was a significant positive correlation between the mean dwell time in state I and the resting tremor score in the PD patients with tremor. This indicated that a higher proportion of time stay in state I was associated with poor level of tremor severity. Compared with the other three states, the strength of FNC between networks in state I was much lower. The fact that PDTD patients cost more time in the weakly connected dFNC state indicates less information communication between networks. This might result in the inability for motor cortex to timely and accurate process information on rapid adaptive top-down control. In addition, as shown in Figure 4D, the average transition matrix showed the probability converts from state *t*−1 to state *t* in color map, and we found that the most frequent state I was as well more likely to transfer across states. Taken together, weak interaction between networks and high variability of transitions reflect state I may be an unstable state, ready to participate in brain activity. Indeed, brain signaling activity fluctuates across different patterns rather than keeping stable overall at the same level of datum line. Our result that long time is spent in the unstable state I in TDPD patients also provides corroborating evidence to the above point.

### Dynamic Functional Network Connectivity Strength Changes Between PDTD and PDNTD Patients

Both PDTD and PDNTD patients displayed higher connectivity between dSMN and FPN in state II and state III compared with the controls. Here, dSMN mainly refers to supplementary motor area and paracentral lobule, relating to planning and execution of voluntary movements (Manara et al., 2018; Rodriguez-Sabate et al., 2019). FPN is a crucial region involving cues signifying task onset and responding differentially to tasks that carry performance feedback (Dosenbach et al., 2008). Functional connectivity between the SMN and FPN may reflect motor performance, and their coupling is found to be bound up with motor outcome in patients with PD and chronic stroke (Lam et al., 2018; Chen et al., 2021). However, our results are inconsistent with a recent study by Chen et al. (2021), who found reduced communication between FPN and SMN in PD. One likely explanation is that the increased dSMN-FPN coupling may present a functional compensation. The PD patients in our study had a short course of disease and were at an early stage, while the disease course in the study by Chen et al. was relatively long. Hence, we presume that the functional connectivity might be repairable in the incipient stage, whereas the impairment of dSMN-FPN coupling became apparent as the disease progressed to an advanced stage.

We found that PDNTD patients exhibited decreased FNC between BG and FPN compared with both PDTD patients and controls. This result is in line with a task-related study (Prodoehl et al., 2013) revealing that PDNTD patients had reduced activity in the globus pallidus and ipsilateral dorsolateral prefrontal cortex, key player in the BG and FPN, respectively, compared with tremor-dominant PD patients and controls. Frontoparietal systems, known as cognitive control networks, play an important role in cognitive task and environment adaption (Cole et al., 2013), and the dynamic property of the FPN is associated with cognitive control (Zanto and Gazzaley, 2013). Decreased FC between BG and FPN indicates that the impairment of cognitive ability might be more severe in PDNTD patients, which further clarifies the point that PDNTD patients have worse cognitive performance than PDTD patients (Burn et al., 2006) and higher risk of developing dementia (Williams-Gray et al., 2007).

Interestingly, we also found a significant increase in the connectivity of l_FPN and r_FPN in PDNTD patients, which supports a previous study exploring interhemispheric functions in PD, described in the alpha1 band oscillatory activity: Interhemispheric coupling was positively associated with cognitive dysfunction in early-stage PD patients, an increased tendency for perseveration (Stoffers et al., 2008). Thus, we speculate that excessive interhemispheric coordination in bilateral FPN regions in PDNTD patients may be the manifestation of an attempt to invest more neural resources to compensate for the cognitive decline.

Tremor-dominant PD patients showed increased FNC between BG and vSMN in state IV compared with PDNTD, and the FNC strength of these areas was positively correlated with the total tremor score. Dysfunction of the basal ganglia-motor circuit is generally believed to underlie the movement disorders in PD (Dirkx et al., 2016). The current dominantly held view is that tremulous activity originates from the basal ganglia and spreads through cerebello-thalamo-cortical circuit to the motor cortex (Helmich et al., 2012; Dirkx et al., 2016; Helmich, 2018). The SMN is critical for the planning and execution of the simplest motor patterns (Smith et al., 2009). Thus, the increase in FNC of PDTD patients between BG and SMN suggested abnormalities in controlling the execution of the motor performances in PDTD patients. There are many previous studies that showed increased connectivity strength and elevated regional hypermetabolism in the basal ganglia and motor cortex in Parkinsonian tremor (Rivlin-Etzion et al., 2008; Mure et al., 2011; Zhang D. et al., 2015). In addition, the increased expression of functional connectivity between networks was similar to the excessive neurosynchrony in cerebral cortex, and changes in the synchronization of brain signals were reported to be well-associated with Parkinsonian tremor (Rivlin-Etzion et al., 2008; Stoffers et al., 2008). In addition to consistency of previous studies, our findings also provide other insights from a dynamic perspective. Furthermore, correlation analysis revealed that the increased BG-vSMN coupling was positively associated with the expression of total tremor level in PDTD patients. In other words, greater the strength of BG-vSMN coupling in state IV, the PD patients would have worse tremor performance measured by MDS-UPDRS tremor subscore. Of note, scores of rest tremor, postural tremor, and kinetic tremor constitute total tremor score. The correlation indicates the effectiveness of the detected alterations in capturing the expressions of tremor severity in PDTD patients. Inspired by these findings, we postulate that the increased FNC between BG and vSMN may lead the basal ganglia to be more influenced by top-down mechanisms, thereby resulting in the loss of segregation in the dopamine-depleted basal ganglia and cause network more instability.

### Higher Variability of Global Efficiency in PDNTD Patients

Furthermore, we observed that PDNTD patients showed higher variability in global network efficiency relative to health controls, suggesting instability and enthusiasm of dynamic interactions. Our finding was in line with the current studies by Kim et al. (2017) and Zhu et al. (2019), which reported that PD patients have higher variability of global network efficiency, suggesting low efficiency and instability of information propagate within/between network. Our results further clarify that the difference of global network efficiency between PD patients and the controls is due to the aberrant information integration of brain networks in PDNTD patients. The result that PDTD patients did not exhibit aberrant global network efficiency may also explain the more benign disease course of PDTD patients. Autopsy and nuclear imaging studies reported that PDTD patients have relatively benign nigrostriatal degeneration (Paulus and Jellinger, 1991), and more cortical lesions were found to exist in PDNTD patients than in PDTD patients. This may be because resting tremor may appear as a side effect of brain mechanisms to make up for the changes that produce other movement disorders (Rivlin-Etzion et al., 2006).

### Distinguishing PD Phenotypes

The univariate ROC analysis shows that the diagnostic value of mean dwell time in state I for PD phenotypes is relatively high. Combined with linear correlation analysis, our study illustrates that the mean dwell time in state I was closely related to the severity of the clinical manifestations. Our study is an attempt for furnishing relevant classification of PD phenotypes and exploring the potential of mean dwell time in state I as a biomarker for PD phenotypes seems valuable. Other than the mean dwell time in state I, PDTD and PDNTD patients also exhibited significant difference in terms of the UPDRS III. However, according to linear correlation analysis, there was no correlation between UPDRS III score and observed dFNC changes when controlling for covariables including sex, age, and head movement. Thus, the observed dFNC changes may not be related to the motor impairment.

## Limitations

Several limitations should be taken into account in our study. First, the sample size was relatively small to evaluate the dFC abnormalities. Thus, a larger number of subjects should be included in the future studies. Second, our study did not include cerebellum that may involve assisting in Parkinsonian tremor genesis. Although the main purpose of this study is to study the key role of the brain in Parkinsonian tremor, it is still advocated to add the cerebellum in order to observe the overall impact in future research studies. Third, it is difficult to control head motion for PD patients without medication, and head motion may bring some confusion to the result of dFC. In order to minimize the impact of head motion, we eliminated three subjects with head motion exceeding 3 mm of translation motion or 3 degrees of rotation and used head motion correction algorithm to adjust the images, and the mean framewise displacement values did not differ among the triple groups in this study. Fourth, our study is indeed unable to elucidate the causal relationship between PD phenotypes and brain dFNC changes. Further research studies are needed to explore the “cause-consequence” relationship.

## Conclusions

Combining ICA and graph theory analysis applied to dFC, our study provides a full map of the presence and difference in abnormal dFC between PD subtypes. Severity of tremor is associated with dwell time in state I and increased FNC between BG and vSMN in state IV in PD patients. These findings provide significance for understanding the neural substrates underlying the PD subtypes and suggest that dFC may serve as a novel physiological biomarker to identify PD subtypes.

## Data Availability Statement

Publicly available datasets were analyzed in this study. This data can be found at: Parkinson's Progression Marker Initiative database (PPMI, www.ppmi-info.org/data).

## Author Contributions

JZ contributed to the methodology, software, validation, and writing (original draft). QZ and QS contributed to the conceptualization, software, formal analysis, and writing (original draft). JL contributed to the investigation, data curation, and formal analysis. SD contributed to the investigation and data curation. CL contributed to the software and data curation. GC contributed to the resources, supervision, and writing (review and editing). All authors contributed to the article and approved the submitted version.

## Funding

This work was supported by the Guangdong Science and Technology Plan Project (2012B031800137) and the Shenzhen Health and Family Planning System Research Project (SZXJ2018076).

## Conflict of Interest

The authors declare that the research was conducted in the absence of any commercial or financial relationships that could be construed as a potential conflict of interest.

## Publisher's Note

All claims expressed in this article are solely those of the authors and do not necessarily represent those of their affiliated organizations, or those of the publisher, the editors and the reviewers. Any product that may be evaluated in this article, or claim that may be made by its manufacturer, is not guaranteed or endorsed by the publisher.
